# Retrospective analysis of 119 Chinese noninflammatory locally advanced breast cancer cases treated with intravenous combination of vinorelbine and epirubicin as a neoadjuvant chemotherapy: a median follow-up of 63.4 months

**DOI:** 10.1186/1471-2407-9-375

**Published:** 2009-10-21

**Authors:** Ou Huang, CanMing Chen, JiaYi Wu, ShuJie Chen, XiaoSong Chen, GuangYu Liu, Zhen Hu, JingSong Lu, Jiong Wu, ZhiMin Shao, ZhenZhou Shen, KunWei Shen

**Affiliations:** 1 Department of Surgery, RuiJin Hospital, School of Medicine, Shanghai Jiaotong University, Shanghai 200025, PR China; 2 Department of Breast Surgery, Cancer Hospital, Fudan University, Shanghai 200032, PR China

## Abstract

**Background:**

This study is a retrospective evaluation of the efficacy of neoadjuvant chemotherapy (NC) with a vinorelbine (V) and epirubicin (E) intravenous combination regimen and is aimed at identification of predictive markers for the long-term outcome in noninflammatory locally advanced breast cancer (NLABC).

**Methods:**

One-hundred-and-nineteen patients with NLABC were identified from September 2001 to May 2006. Analysis was performed in March 2008, with a median follow-up of 63.4 months (range, 9-76 months). All patients were diagnosed with invasive breast cancer using 14 G core needle biopsy and treated with three cycles of VE before surgery. Local-regional radiotherapy was offered to all patients after the completion of chemotherapy followed by hormonal therapy according to hormone receptor status. Tissue sections cut from formalin-fixed paraffin-embedded blocks from biopsy specimens and postoperative tumor tissues were stained for the presence of estrogen receptor (ER), progesterone receptor (PgR), HER-2 (human epidermal growth factor receptor-2), and MIB-1(Ki-67).

**Results:**

Patients characteristics were median age 52 years (range: 25-70 years); clinical TNM stage, stage IIB (n = 32), stage IIIA (n = 56), stage IIIB (n = 22) and stage IIIC (n = 9). All patients were evaluable for response: clinically complete response was documented in 27 patients (22.7%); 78 (65.6%) obtained partial response; stable disease was observed in 13 (10.9%); 1 patient (0.8%) had progressive disease. Pathological complete response was found in 22 cases (18.5%). Seventy-five patients were alive with no recurrence after a median follow-up of 63.4 months, the 5-year rates for disease-free survival and overall survival were 58.7% and 71.3%, respectively, after the start of NC. On multivariate analysis, the independent variables associated with increased risk of relapse and death were high pre-Ki-67(p = 0.012, p = 0.017, respectively), high post-Ki-67 expression (p = 0.045, p = 0.001, respectively), and non-pCR (p = 0.034, p = 0.027, respectively). A significantly increased risk of death was associated with lack of pre-ER expression (p = 0.002). Among patients with non-pCR, those with a pathological response at the tumor site with special involvement (i.e. skin, vessel and more than one quadrant) were at a higher risk of disease relapse and death (p < 0.001, p = 0.001, respectively).

**Conclusion:**

This study suggests the promising use of a VE regimen as NC for Chinese NLABC after a median follow-up of 63.4 months. Pathological response in the tumor site, pre-Ki-67 and post-Ki-67 expression, and pre-ER expression were the important variables that predicted long-term outcome. Patients with pathological special involvement at the primary site after NC had the lowest survival rates.

## Background

Locally advanced breast cancer (LABC) comprises a heterogeneous group of breast neoplasms, from stages IIB to IIIC according to the American Joint Committee on Cancer (AJCC) staging system [1]. These cancers are distinct from other breast cancers in terms biological characteristics and clinical behavior, showing aggressive behavior and highly angiogenic characteristics. Neoadjuvant chemotherapy (NC) or primary chemotherapy is at present the standard therapy for LABC, and an increasingly popular treatment strategy for operable breast cancers [2-7]. NC allows regression of the tumor in order to avoid mastectomy and to eliminate clinically undetectable micrometastases. In addition, NC permits the assessment of the response of the primary tumor to a particular chemotherapy regimen and provides an early opportunity to change therapeutic agents if the tumor appears clinically resistant.

A series of anthracyclines and vinorelbine combination in advanced or metastatic breast cancer was reported during the 1990s [8-12]. Only one phase III randomized trial of MA8, conducted by the National Cancer Institute of Canada (NCIC) in 2000, compared single-agent doxorubicin with the combination of vinorelbine plus doxorubicin in metastatic breast cancer, and failed to show any added effect for the combination[9]. However, before 2001, there were no published clinical trials of epirubicin-vinorelbine based combinations for neoadjuvant treatment in LABC. We conducted a phase II prospective clinical trial of vinorelbine and epirubicin (VE) as a NC regimen in the treatment of Chinese LABC at the Cancer Hospital of Fudan University from September 2001 to December 2004; this study was approved by the institutional review board of my institution [13]. Based on the positive results of this regimen and the absence of a standard chemotherapy regimen for LABC in China at the time, some patients with LABC continued the treatment after the completion of the study. These patients were informed of the risks and benefits of the treatment, and provided written informed consent. Since the prognoses of noninflammatory locally advanced breast cancer (NLABC) and inflammatory breast cancer (IBC) are different, despite similar treatment regimens[14], we retrospectively analyzed the data of NLABC patients who received VE as a neoadjuvant chemotherapy regimen from September 2001 to May 2006 at our institution. We evaluated the efficacy of VE and the impact of clinical, pathological, and immunohistochemical features of breast cancer on survival after a median follow-up of 63.4 months in order to identify biomarkers that may effectively predict the long-term outcome of this combination regimen in NLABC.

## Methods

### Patient Selection

From September 2001 to May 2006, 119 patients with NLABC who had been treated with three cycles of NC with intravenous VE regimen and without other local or systemic treatment before surgery were identified at the Cancer Hospital of Fudan University in China. The VE regimen consisted of 25 mg/m^2 ^of intravenous V given on day 1 and on day 8 plus 60 mg/m^2 ^of E given on day 1 and then every 21 days. Other eligibility criteria for the this study met by all patients were being female, 18-70 years of age, no other malignant tumors or tumor history, diagnosis with invasive breast cancer via 14 G core needle biopsy, lack of metastatic spread as excluded by chest X-ray and abdominal ultrasound before or after initial diagnosis, and adequate cardiac, renal and hepatic function as well as normal marrow reserves. The baseline work-up before neoadjuvant chemotherapy included a complete history and clinical examination, bilateral mammography, bilateral breast and axillary ultrasound, and percutaneous cytology of lymph nodes by fine-needle aspiration (FNA) to assess potential clinically suspicious lymph node involvement.

All patients gave written informed consent and the study protocol was designed according to the principles of the Helsinki guidelines and approved by the institutional review board of my institution.

Toxicity was assessed through clinical or laboratory examination, or through other special examinations such as EKG if considered necessary before each cycle of chemotherapy and surgery. A complete blood cell count was repeatedly performed every 2-4 days during chemotherapy in order to detect hematologic side effects. World Health Organization (WHO) criteria were applied to evaluate the toxicities of the neoadjuvant chemotherapy

### Treatment

After full assessment of response and restaging within 2 weeks from the last NC session, all patients were subjected to surgery within 4 weeks. Because the breast-conserving therapy is not widely accepted by Chinese patients, accounting for less than 10% of operable breast cancers as reported by Zhang[15], all patients in our study chose modified radical mastectomy or radical mastectomy if the residual tumor invaded the pectoralis major muscle. Subsequently, patients received three cycles of VE as adjuvant chemotherapy after surgery, if clinical complete response (cCR) or clinical partial responses (cPR) were achieved. The FEC regimen, consisting of 500 mg/m2 of intravenous 5-fluorouracil, 75 mg/m2 of epirubicin and 600 mg/m2 of cyclophosphamide given on day1 and then every 3 weeks for 3 cycles was administered in patients with clinically stable disease (cSD) and clinically progressive disease (cPD). All patients received a total of six chemotherapy cycles.

Local-regional radiotherapy was offered to all patients within 4 weeks after the completion of adjuvant chemotherapy and was given over 5 weeks with a total dose of 50-60 Gy in 1.8-2.0 Gy daily fractions to the breast, internal mammary lymph nodes, and supraclavicular axillary lymph node areas. Hormonal adjuvant therapy was offered to all ER or PR positive patients before exposure to NC or after surgery; this consisted of tamoxifen for premenopausal patients and aromatase inhibitors for postmenopausal patients. Hormonal adjuvant therapy was started at the end of adjuvant treatment.

### Assessment of Response

Clinical, mammographic, and ultrasound measurements were recorded before treatment and at the end of the cytotoxic treatment before surgery. The clinical assessment of response was based on measurements of the longest diameter of the tumor and node according to the standard of RECIST (response evaluation criteria in sold tumors)[16], and was classified as follows: clinical complete response (cCR), the disappearance of disease; clinical partial response (cPR), at least a 30% decrease; clinical progressive disease (cPD), at least a 20% increase in the sum of the longest diameter of target lesions or the appearance of new lesions; clinical stable disease (cSD), neither sufficient shrinkage to qualify for cPR nor sufficient increase to qualify for cPD.

The pathological response was evaluated after surgical resection of the remaining tumor. Pathologic response in the primary site (pRT) was classified as follows: T-A) no tumor cell or presence of in situ carcinoma, no invasive tumor; T-B) presence of invasive carcinoma without special involvement (i.e., skin, vessel, more than one quadrant); T-C) presence of invasive carcinoma with special involvement(i.e., skin, vessel, more than one quadrant). A pathological response was considered complete (pCR) if no residual or in situ tumor could be detected (pCR = T-A, non-pCR = T-B+ T-C).

### Immunohistochemistry and Scoring methods

Tissue sections were cut from formalin-fixed paraffin-embedded blocks prepared from biopsy specimens and postoperative tumor tissue. Sections containing representative tumor samples were assayed by immunohistochemistry for the presence of the estrogen receptor (ER), progesterone receptor (PgR), HER-2 (human epidermal growth factor receptor-2), and MIB-1(Ki-67). Immunohistochemistry and the scoring of immunohistochemical staining of ER, PR and Her-2/neu were carried out in the pathology department of our hospital. Staining results were assessed by at least two pathologists using a semiquantitative scoring system, and the final score was calculated as the product of a proportion score and an intensity score. The proportion score, indicating the percentage of tumor cells stained, was interpreted as follows: a score of 0 represented no staining seen, one represented ≤ 25% of cells positive, two represented 25-50% of cells stained, three represented 50-75% of positive cells and four represented >75% of cells showing positive staining. According to the intensity score, a negative result was defined as a score of 0, weakly positive as one, moderately positive as two, and strongly positive as three. The staining results therefore ranged from score 0 to 12. The scoring system for ER and PR was defined as negative for a score of 0 and positive for scores of 1 through 12 based on the nucleic staining of carcinoma cells. Her-2/neu staining was defined as negative for scores of 0-8 (namely, 0, 1+ and 2+ in the DAKO scoring system) and positive for a strong membranous staining with scores of 9-12 (namely DAKO score 3+). The Ki-67 score was counted in a minimum of 10 randomly selected 40× high-power fields containing representative sections of tumor tissue and calculated as the percentage of positively stained cells as compared to total cells.

### Statistical Analysis

Statistical analyses were performed using SPSS statistical software package 15.0. Relationships between dichotomous variables were analyzed using a chi-square test or Fisher's exact test, where appropriate. P-values < 0.05 were considered significant. Ki-67 as a continuous variables was defined as high expression (>20%) or low expression (≤ 20%) according to their median expression level before NC. Results were last updated in March 2008. A total of 109 patients were evaluated with the paired correlation before and after surgery, the other 10 patients showed no residual tumor in the postoperative tissue. Disease-free survival (DFS) was defined as the time elapsed between the date of the start of NC and the date of first time relapse, wherever this relapse might be. Death in patients without a record of relapse was counted as an event for disease-free survival. Overall survival (OS) was the time between the date of initial diagnosis and date of last status report, with the patient being alive or dead and regardless of the cause of death. Survival analyses were performed using the Kaplan and Meier method, and the differences between groups with regard to survival time were evaluated by the log-rank test. Univariate analyses (log-rank tests) and multivariate analyses (Cox regression analyses) were performed to identify risk factors associated with DFS and OS.

## Results

### Patient characteristics before and after chemotherapy

From September 2001 to May 2006, one hundred and nineteen patients with NLABC were identified. The median age at diagnosis was 52 years (range, 25 -70 years) and 50 (42%) of these women were younger than 50 years. Only one patient with disease progression after two cycles of primary VE combination was submitted to local treatment. The distribution of clinical involvement showed that all the patients had tumors >2.0 cm. The characteristics of all patients before and after surgery were summarized in Table 1.

**Table 1:** Patient Characteristics before and after Surgery (n = 119)

Characteristics	No.(%)	Characteristics	No.(%)
Clinical stage		Grade	
II B	32 (26.9%)	I	1 (0.8%)
III A	56 (47.1%)	II	67 (56.3%)
III B	22 (18.5%)	III	15 (12.6%)
III C	9 (7.5%)	Not graded	36 (30.3%)
Pre-cALN		Post-cALN	
negative	36 (30.3%)	negative	81 (68.1%)
positive	83 (69.7%)	positive	38 (31.9%)
Pre-ER		Post-ER※	
negative	58 (48.7%)	negative	82 (75.2%)
positive	61 (51.3%)	positive	27 (24.8%)
Pre-PR		Post-PR※	
negative	60 (50.4%)	negative	84 (77.1%)
positive	59 (49.6%)	positive	25 (22.9%)
Pre-HER-2		Post-HER-2※	
0-2+	86 (72.3%)	0-2+	79 (72.5%)
3+	33 (27.7%)	3+	30 (27.5%)
Pre-Ki67		Post-Ki67※	
≤ 20%	74 (62.2%)	≤ 20%	93 (85.3%)
>20%	45 (37.8%)	>20%	16 (14.7%)
Pathology		Pathological tumor size	
IDC	92 (77.3%)	≤ 2 cm	28 (23.5%)
ILC	4 (3.4%)	> 2 cm	59 (49.6%)
other	23 (19.3%)	unmeasured	32 (26.9%)
Clinical response		pALN	
CR	27 (22.7%)	N 0	39 (32.8%)
PR	78 (65.6%)	N 1-3	24 (20.2%)
SD	13 (10.9%)	N 4-9	35 (29.4%)
PD	1 (0.8%)	N >9	21 (17.6%)
Pathological response in tumor site			
T-A	22 (18.5%)		
T-B	75 (63.0%)		
T-C	22 (18.5%)		

Abbreviations: pre-, before chemotherapy; post-, after chemotherapy; cALN, clinical axillary lymph node; pALN: pathological axillary lymph node; IDC: Invasive ductal cancer; ILC: Invasive lobular cancer; CR, Complete response; PR, Partial response; SD, Stable disease; PD, Progressive disease; T-A: no tumor cell or presence of in situ carcinoma, no invasive tumor; T-B: presence of invasive carcinoma without special involvement (such as skin, vessel, more than one quadrant); T-C: presence of invasive carcinoma with special involvement.

※109 patients were evaluated after neoadjuvant chemotherapy, because of 10 patients with no residual tumor in postoperative tissue.

### Clinical Response and Pathological Evaluation

As shown in Table 1, clinical evaluation of the treatment efficacy indicated overall responses in 105 of the 119 patients (88.3%), with 27 (22.7%) cCR, and 78 (65.6%) cPR.

A total of 117 patients underwent modified radical mastectomy, and 2 patients had radical mastectomy. All patients received examination of over 10 axillary lymph nodes. The pCR in the breast was achieved in 22 patients (18.5%) and in the axillary lymph nodes in 39 patients (32.8%), while absence of tumors in the breast occurred in 10 patients (8.4%). For patients who experienced a pCR, 68.2% of the tumors were pre-ER negative and 31.8% pre-ER positive. Pre-ER negative patients were more likely to achieve pCR after NC (p = 0.043, Table 2).

**Table 2:** Relations between pre-ER and Pathological Response in Tumor Site

Pathological Response in Tumor Site	pre-ER			p value
		
	negative	positive	total	
pCR	15(68.2%)	7(31.8%)	22(100%)	0.043
non-pCR	43(44.3%)	54(55.7%)	97(100%)	
total	58	61	119	

Abbreviations: pCR: pathological complete response; pre-ER:ER status examined before neoadjuvant chemotherapy.

At surgery, the median number of involved nodes was 4 (range, 0-28). Among 80 patients who showed pathological evidence of node involvement after surgery, 24 (20.2%) were N1-3, 35 (29.4%) were N4-9, and 21 (17.6%) were N>9. 83 of 119 patients had percutaneous cytological evidence of node involvement before neoadjuvant chemotherapy. Among these patients, pathological nodes were found in 59 of 83 patients (71.1%). Pathological nodes were found in 21 of 36 patients (58.3%) without clinical node involvement or for whom percutaneous cytology was negative disease before neoadjuvant chemotherapy.

### Biomarker expression

The evaluation of ER, PR, Ki-67, and HER-2 protein expression was possible in 119 patients before NC and in 109 patients after NC, because the postoperative tissue did not contain tumor cells for analysis in 10 patients. Positive ER expression was detected in 61 of 119 patients (51.3%) before and in 27 of 109 patients (24.8%) before and after surgery, respectively. The expression of PR protein was positive in 59 patients (49.6%) and 25 patients (22.9%) before and after surgery, respectively. High HER-2 protein expression was observed in 33 patients (27.7%) before NC. After NC, high expression of HER-2 protein was observed in 30 patients (27.5%). The median and range of Ki-67 indices before NC and after surgery were 21.9% (0%-67%), and 11% (0%-62%), respectively. We recorded the Ki-67 as high expression (>20%) and low expression (≤ 20%). High Ki-67 protein expression was observed in 45 patients (37.8%) before NC. After NC, high expression of Ki-67 protein was observed in 16 patients (14.7%). Table 1 shows immunohistochemical protein expression levels (ER, PR, HER-2, and Ki-67) before (pre-)and after (post-)surgery.

### Toxicity of the regimens

The combination of vinorelbine and epirubicin was well tolerated with no case of death due to drug-related toxicity. The toxicities associated with therapy are summarized in Table 3. Neutropenia was the major hematologic toxicity. Grade 3-4 neutropenia was observed in 55 patients (46.2%) and 27.3% of chemotherapy induction cycles. Febrile neutropenia was found in 16 patients (13.4%) but only 5 patients (4.2%) required hospitalization. Significant anemia and thrombocytopenia associated with therapy were very scarce. The most common nonhematologic toxicity was alopecia and nausea/vomiting. Grade 3 alopecia was seen in 63.6% of patients. Almost all patients experienced different degrees of nausea/vomiting after the administration of chemotherapy and grade 3 nausea/vomiting was found in 12.6% patients. Five patients experienced grade 3 mucositis. The other nonhematologic toxicities were minimal and clinically insignificant.

**Table 3:** Grade 3-4 toxicity

Toxicity	**No**.	%
Neutropenia	55	46.2%
Anemia	---	---
Thrombocytopenia	2	1.7%
Alopecia	76	63.9%
Nausea/vomiting	15	12.6%
Mucositis	5	4.2%
Hepatic function	3	2.5%
Neuropathy	---	---
Cardiac function	---	---

### Follow-up and Survival

The study was analyzed in March 2008, with a median follow-up of 63.4 months (range, 9-76 months). 115 patients were evaluable for survival (4 patients were lost to follow-up). After the median follow-up, the 5-year rates for DFS and OS were 58.7% and 71.3%, while 6-year rates for DFS and OS were 55.0% and 68.2%, respectively, after the start of chemotherapy. Of the 43 patients who had relapsed, 2 had local recurrence, 6 had local and metastatic recurrence, and 35 had contralateral or metastatic recurrence. Of the 28 patients who died, 22 died as a result of liver or lung failure due to metastasis.

We evaluated the impact of clinical (age, clinical stage before NC, clinical ALNs status before and after surgery, clinical response), pathological (histological grade, tumor size, pathology, axillary lymph node status, and pathological response) and immunohistochemical (ER, PR, Ki-67 and HER-2 protein expression before and after surgery) features on disease free survival (DFS) and overall survival (OS).

As table 4 shows, univariate analysis of pCR (p < 0.001, p = 0.003, respectively) and low post-Ki-67expression (p = 0.025, p = 0.001, respectively) revealed association with significantly decreased risk of relapse and death. A significantly increased risk of death was associated with lack of pre-ER or pre-PR expression (p = 0.045, p = 0.026, respectively). There was no relationship between clinical response (p = 0.284, p = 0.651, respectively) and pathological axillary lymph node status (p = 0.456, p = 0.425, respectively) with DFS and OS.

**Table 4:** Relations between pre-ER and Pathological Response in Tumor Site

	DFS		OS	
	
Variables	No.(%)	Log-Rank P	No.(%)	Log-Rank P
Before surgery				
Age				
≤ 50	30(41.7%)		36(41.4%)	
>50	42(58.3%)	0.792	51(58.6%)	0.638
Clinial stage				
II B	20(27.8%)		23(26.4%)	
III A	34(47.2%)		39(44.9%)	
III B	14(19.4%)		18(20.7%)	
III C	4(5.6%)	0.517	7(8.0%)	0.195
cALN				
negative	21(29.2%)		25(28.7%)	
positive	51(70.8%)	0.522	62(71.3%)	0.295
ER				
negative	33(45.8%)		37(42.5%)	
positive	39(54.2%)	0.580	50(57.5%)	0.045
PR				
negative	32(44.4%)		38(43.7%)	
positive	40(55.6%)	0.155	49(56.3%)	0.026
HER-2				
0-2+	52(72.2%)		63(72.4%)	
3+	20(27.8%)	0.778	24(27.6%)	0.643
Ki67				
≤ 20%	49(68.1%)		58(66.7%)	
>20%	23(31.9%)	0.071	29(34.3%)	0.055
Pathology				
IDC	57(79.1%)		69(79.3%)	
ILC	2(2.8%)		2(2.3%)	
other	13(18.1%)	0.987	16(18.4%)	0.934
Clinical response				
CR	20(27.8%)		11((12.6%)	
PR	44(61.1%)		54(62.2%)	
SD	7(9.7%)		21(24.1%)	
PD	1(1.4%)	0.284	1(1.1%)	0.651

After surgery				
cALN				
negative	49(68.1%)		60(69.0%)	
positive	23(31.9%)	0.848	27(31.0%)	0.912
ER※				
negative	55(76.4%)		67(77.0%)	
positive	17(23.6%)	0.965	20(23.0%)	0.827
PR※				
negative	54(75.0%)		67(77.0%)	
positive	18(25.0%)	0.428	20(23.0%)	0.864
HER-2※				
0-2+	50(69.4%)		60(69.0%)	
3+	22(30.6%)	0.568	27(31.0%)	0.328
Ki67※				
≤ 20%	66(91.7%)		80(92.0%)	
>20%	6(8.3%)	0.025	7(8.0%)	0.001
pALN				
N 0	23(31.9%)		27(31.0%)	
N 1-3	18(25.0%)		20(23.0%)	
N 4-9	18(25.0%)		23(26.5%)	
N >9	13(18.1%)	0.456	17(19.5%)	0.425
Pathological tumor size				
≤ 2 cm	14(19.4%)		18(20.7%)	
> 2 cm	41(56.9%)		48(55.2%)	
unmeasured	17(23.7%)	0.256	21(24.1%)	0.303
Grade				
I	1(1.4%)		1(1.2%)	
II	39(54.2%)		47(54.0%)	
III	8(11.1%)		11(12.6%)	
Not graded	24(33.3%)	0.624	28(32.2%)	0.827
pathological response in tumor site				
pCR	21(29.2%)		22(25.3%)	
non-pCR	51(70.8%)	<0.001	65(74.7%)	0.003

Abbreviations: DFS, disease free survival, OS, overall survival; OR, odds ratio; 95%CI, 95% confidence interval; cALN, clinical axillary lymph node; pALN: pathological axillary lymph node; IDC: Invasive ductal cancer; ILC: Invasive lobular cancer; CR, complete response; PR, partial response; SD, stable disease; PD, progressive disease; pCR, pathological complete response.

※109 patients were evaluated after neoadjuvant chemotherapy, because of 10 patients with no residual tumor in postoperative tissue.

*115 Patients were evaluable for survival (4 patients were lost to follow-up)

On multivariate analysis, high pre-Ki-67 (p = 0.012, p = 0.017, respectively) and post-Ki-67 expression (p = 0.045, p = 0.001, respectively), and non-pCR (p = 0.034, p = 0.027, respectively) were independent variables associated with the increased risk of relapse and death (Table 5).

**Table 5:** Multivariate analyses of characteristics before and after surgery in 115 patients as predictors of relapse and death

Variables	DFS			OS		
	
	HR	95% CI	P value	HR	95% CI	P value
pre-ER			NS	0.271	0.119-0.619	0.002
pre-Ki-67	2.239	1.191-4.207	0.012	2.668	1.192-5.974	0.017
pos-Ki-67	2.174	1.019-4.637	0.045	4.646	1.918-11.258	0.001
pRT	3.588	1.104-11.660	0.034	9.796	1.304-73.567	0.027

pRT, pathological response in tumor site;95%CI, 95% confidence interval; HR, hazard ratio; NS, no significance.

**Figure 1:**
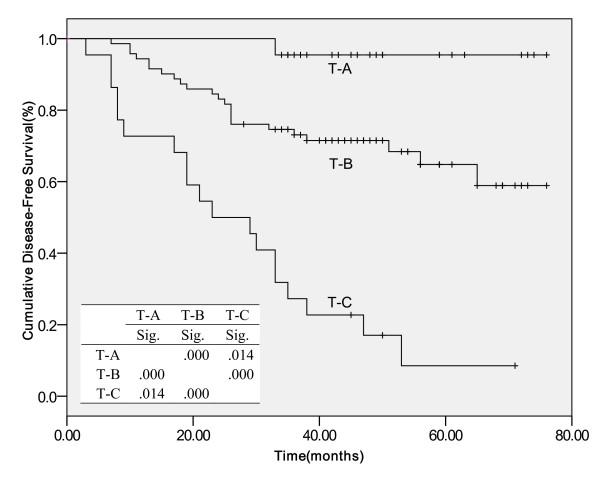
**Cumulative Disease-Free Survival**. Kaplan-Meier survival estimates according to pathological response in tumor site Abbreviations: T-A, no tumor cell or presence of in situ carcinoma, no invasive tumor; T-B, presence of invasive carcinoma without special involvement (such as skin, vessel, more than one quadrant); T-C, presence of invasive carcinoma with special involvement; Sig, significance. *Table within the figure was the log-rank P value between two curves.

**Figure 2:**
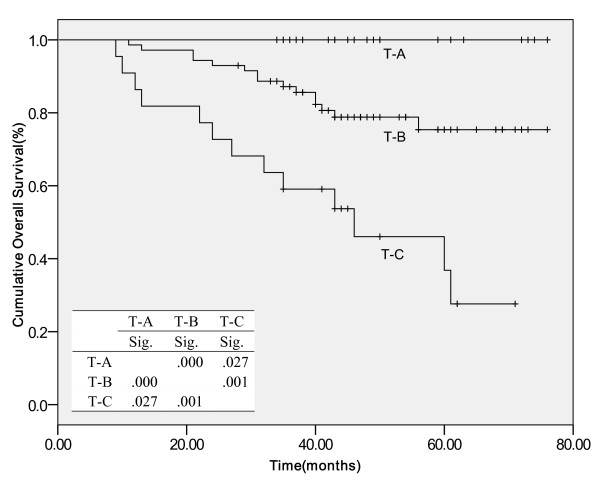
**Cumulative Overall Survival**. Kaplan-Meier survival estimates according to pathological response in tumor site Abbreviations: T-A, no tumor cell or presence of in situ carcinoma, no invasive tumor; T-B, presence of invasive carcinoma without special involvement (such as skin, vessel, more than one quadrant); T-C, presence of invasive carcinoma with special involvement; Sig, significance. *Table within the figure was the log-rank P value between two curves.

Compared to other pathological responses in the tumor site, patients with special involvement had the worst survival rates, indicating that more effective adjuvant chemotherapy may be considered for treatment of these patients. Figures 1 and 2 showed the Kaplan-Meier curve for DFS and OS according to the pathological response in the tumor site.

## Discussion

LABC remains a major clinical problem despite significant progress made in the treatment. Despite of a multi-disciplinary approach including surgery, chemotherapy, endocrine therapy and radiation therapy, most patients with LABC have a poor prognosis. NC is now the standard therapy for LABC and an increasingly popular treatment strategy for operable breast cancer. Several landmark clinical trials have shown that NC is at least as effective as post-operative adjuvant chemotherapy with the same regimen in terms of disease-free and overall survival in operable breast cancer [17-19].

In order to find predictors for survival after NC in primary tumors, we assessed clinical and tumor biologic parameters that were measured before and after NC in 119 NLABC patients treated with VE. Although breast conservation surgery after NC was offered and its advantages were explained by surgeons, all patients with LABC in our study chose modified radical mastectomy or radical mastectomy if the residual tumor invaded the pectoralis major muscle. IBC and LABC are both locally advanced breast carcinomas with poor prognosis, and are often grouped together in clinical trials. However, IBC and LABC are distinct clinicopathologic entities with different prognostic factor profiles including age-specific incidence rate patterns, presence of key pathways and protein expression[14]. To avoid potential confounding factors, IBC was excluded from our study.

In our study, the clinical overall response rate (cCR+cPR) was 88.3%, with pCR in 18.5% of patients. With a median follow-up of 63.4 months, the 5-year rate for DFS and OS was 58.7% and 71.3%. The results are consistent with other reports for the vinorelbine-containing neoadjuvant chemotherapy, where the clinical response rate varies from 42% to 98%, and the pCR ranges from 4.2% to 32% [20-25]. In comparison to the results of NSABP B-27 as reported by Bear et al. [26], the VE regimen in our study reached a higher pCR rate than the AC regimen (12.6%), and a lower rate than AC following docetaxel treatment(26.1%). The 5-year DFS and OS were inferior to AC or AC following docetaxel arm in the Bear study. These differences in results could be due to the fact that all patients had LABC in our study, while only patients with primary breast tumors larger than 1 cm in diameter were included in the study by Bear[27].

It is generally held that the tumor response to neoadjuvant chemotherapy should be strongly correlated with the long-term outcome of patients. Fisher et al. [17] reported that women whose tumors were no longer clinically evident after NC had a better outcome than those whose tumors showed a cPR to therapy. The latter fared only marginally better than women whose tumors had a cSD and cPD, even though all tumors designated as cPR decreased in size by more than 50% in their study. However, in the multivariate analysis of our study, the clinical response was not predictive of DFS or OS. We assume that the shrinkage of primary tumors after NC may not completely represent the intrinsic nature of the malignant tumors. As reported in the literature [26,28-30], tumors with relatively poor prognostic factors are more sensitive to NC and more likely to achieve pCR. This type of tumor tends to respond initially to chemotherapy but then relapsed rapidly, while relatively low-risk tumors do not show a marked response but progress rather slowly. In addition, the patients included in this study had advanced disease(IIB※IIIC), which may also account for the lack of difference in survival based on response to therapy. Clinical tumor response was assessed by palpation, which is a rather crude and subjective measure for tumor regression.

The pathological response to NC, as determined by an individual in vivo chemo-sensitivity test, reflects tumor biology more accurately and might be a better predictor of outcome. Pathological complete response (pCR) has been confirmed to be associated with prolonged disease-free and overall survival [31,32], and thus has been used as the primary endpoint instead of OS in clinical trials. In our study, all patients achieving pCR were alive after a median follow-up of 63.4 months, except for one patient who had local recurrence. Compared to non-pCR patients, those with pCR had significantly lower risk of recurrence or death on univariate and multivariate analysis. As other studies have reported[26,28-30], we found that those patients who were ER negative patients before treatment were more likely to achieve pCR than ER positive patients.

The pCR as a pure surrogate end point still has its limitations. In the latest report of a 16-year follow-up of the NSABP B27 study[26], the addition of neoadjuvant docetaxel to the AC regimen resulted in a doubled pCR rate but not in an increased survival rate, suggesting that pCR of the breast may not completely take the place of OS as the surrogate end point for neoadjuvant chemotherapy studies. Generally, a large number of patients do not achieve pCR of the breast after NC. This group of patients also has divergent outcomes due to the histological heterogeneity of their breast cancer and is not deemed to have a worse outcome. It is therefore of great importance to identify other predictors of long-term outcome in addition to pCR of the breast.

ALN status has emerged as an important predictor of long-term survival in adjuvant chemotherapy. We expected ALN status before NC and pathologic axillary node involvement to be related to survival. However, we did not find a significant correlation between lymph node status and patients outcome. The prognostic impact of ALNs might be substantially diminished after NC for LABC patients. This result is inconsistent with the finding of Hennessy et al. [32]. These discordant results may be partly explained by the differences in drugs and dosages received by the different patients in each study. Moreover, we failed to find any relationship between pathologic axillary node status and pathological response of the tumors after NC. The biological behaviors of tumor cells appeared to be quite different between primary site and remote sites. However, we cannot exclude the possibility that the limited number of samples included in the analysis may have influenced the results.

The present study investigated the potential relationships between several biomarkers (ER, PR, HER-2 and Ki-67) and the long-term outcome of patients. Hormone receptor status has proven to be among the most important predictive markers for selection of systemic therapy, but its prognostic value, is less clear. In our study, ER and PR status before chemotherapy could prognosticate the OS by univariate analysis. However, the prognostic value of ER status no longer existed after chemotherapy or when evaluated with multivariate analysis. A possible explanations for the weakened prognostic power of ER is that the ER status may change during the course of NC; this has been confirmed by many other researchers [7,33,34], suggesting the pre-treatment ER status might be more reliable for predicting long-term outcome. Moreover, the association of ER/PR status with other established indicators of favorable prognosis such as pCR (more ER negative patients than positive in pCR), and the use of adjuvant endocrine therapies may have decreased the power of the multivariate analysis.

Over-expression of HER-2 is associated with poor outcomes in operable breast cancer. We failed to find any relationship between pre- or post- HER-2 status and survival by univariate and multivariate analysis. This might be explained by the poor outcome of LABC and limited impact of HER-2 in this special group. We also found that the lower pre- and post-Ki-67 expression (Ki-67 ≤ 20%) were significantly correlated with better DFS and OS by multivariate analysis. This finding supports the hypothesis that rapidly growing tumors tend to have a poor prognosis but are also more sensitive to chemotherapy, especially to DNA structure-damaging agents like anthracyclines. Honkoop et al. [35,36] and some other studies [37-41] have demonstrated the prognostic value of the Ki-67 index.

When evaluating the toxicity of therapy, the NE regimen in this study was considered to be well tolerated. There was no septicemia, serious cardiovascular toxicity or side effect-related death. Neutropenia was the major hematologic toxicity. Grade 3-4 neutropenia presented in 46.2% patients enrolled but only 4.2% required hospitalization. Affected patients recovered form neutropenia through support of G-CSF and subsequent therapy was not influenced by the previous neutropenia. Other hematologic toxicities including anemia and thrombocytopenia were infrequent. The most common nonhematologic toxicities included alopecia, nausea/vomiting and mucositis. Patients could tolerate the nonhematologic toxicities and the therapeutic process was not greatly impacted.

## Conclusion

In our experience with a median follow-up of 63.4 months, NC with vinorelbine and epirubicin intravenous combination regimen is a promising treatment for noninflammatory locally advanced breast cancer. In this study, pathological response in tumors, high pre-Ki-67 and post-Ki-67 expression, and pre-ER expression were the important variables that predicted long-term outcome. Patients with pathological special involvement in the primary site after NC showed the worst survival rate, and therefore deserve special attention in the future.

## Competing interests

There is no conflict of interest that could be perceived as prejudicing the impartiality of the research reported. This research was supported by Leading Academic Discipline Project of Shanghai Municipal Education Commission, Project Number: J50208.

## Authors' contributions

OH and CMC carried out the conception and design of the study. KWS participated in the design and administrative support for the study. All authors participated in the provision of patients. OH, CMC, JYW carried out the collection and assembly of data. OH, CMC and KWS participated in the data analysis and interpretation of the study, drafted the manuscript. All authors read and approved the final manuscript.

## Pre-publication history

The pre-publication history for this paper can be accessed here:

http://www.biomedcentral.com/1471-2407/9/375/prepub
